# Pelagic shrimp play dead in deep oxygen minima

**DOI:** 10.1371/journal.pone.0207249

**Published:** 2018-11-28

**Authors:** Benjamin P. Burford, Kyra L. Schlining, Kim R. Reisenbichler, Bruce H. Robison

**Affiliations:** 1 Department of Biology, Hopkins Marine Station, Stanford University, Pacific Grove, California, United States of America; 2 Monterey Bay Aquarium Research Institute, Moss Landing, California, United States of America; University of Connecticut, UNITED STATES

## Abstract

Pelagic crustaceans are arguably the most abundant group of metazoans on Earth, yet little is known about their natural behavior. The deep pelagic shrimp *Hymenopenaeus doris* is a common decapod that thrives in low oxygen layers of the eastern Pacific Ocean. When first observed in situ using a remotely operated vehicle, most specimens of *H*. *doris* appeared dead due to inactivity and inverted orientation. Closer inspection revealed that these animals were utilizing small, subtle shifts in appendage position to control their orientation and sink rate. In this mode, they resembled molted shrimp exoskeletons. We hypothesize that these shrimp may avoid capture by visually-cued predators with this characteristic behavior. The low metabolic rates of *H*. *doris* (0.55–0.81 mg O_2_ kg^-1^ min^-1^) are similar to other deep-living shrimp, and also align with their high hypoxia tolerance and reduced activity. We observed similar behavior in another deep pelagic decapod, *Petalidium suspiriosum*, which transiently inhabited Monterey Canyon, California, during a period of anomalously warm ocean conditions.

## Introduction

The pelagic environment is the largest inhabitable space on the planet, and in many regions contains deep hypoxic layers occupied by animals that are adapted to low oxygen conditions [1]. Many such resident ectotherms exhibit depressed activity levels and metabolic rates [2–6], characteristics that additionally manifest in some transient inhabitants when they descend into deep hypoxic water [7–10]. Although these behavioral and metabolic features are believed to reflect the limited visual predator-prey interactions that prevail under the low light regime of this habitat [11, 12], diffuse irradiance and abundant bioluminescence enable some visually-cued predators to forage in the deep pelagic [1, 12, 13]. In response, visual mimicry is utilized by a variety of resident animals to reduce predation [14, 15]. Pelagic crustaceans are arguably the most abundant group of metazoans on Earth [16]. Although in situ research has examined their vertical movements [17, 18], responses to light stimuli [18, 19], and symbiotic relationships with gelatinous zooplankton [20], little is known about behaviors related to predator evasion under low oxygen conditions in their natural habitat.

The deep pelagic shrimp *Hymenopenaeus doris* is a common decapod endemic to the eastern Pacific Ocean [21–25], that thrives in low oxygen conditions. Under typical climate periods, the northeastern extent of its range, the Gulf of California, Mexico, is characterized by an upwelling ecosystem analogous to that of the California Current, albeit warmer [26, 27]. During anomalously warm climate periods, such as El Niño, upwelling is reduced [28] and increased temperature and hypoxia are evident at depth [26]. The Gulf of California hosts abundant visual predators of penaeoid crustaceans [29] that routinely undertake vertical migrations into oxygen minimum layers [30].

We documented the behavior of *H*. *doris* in situ during a warm climate period in the Gulf of California [28] using a remotely operated vehicle (ROV), and investigated its metabolism through shipboard respirometry measurements on captured specimens. We found this species to exhibit a distinct behavior characterized by inverted orientation and low activity levels that we hypothesize involves mimesis. Through similar methods, we documented comparable behavior in another deep pelagic decapod, *Petalidium suspiriosum*, which transiently inhabited the California Current, during a period of anomalously warm ocean conditions.

## Results and discussion

**Fig 1:**
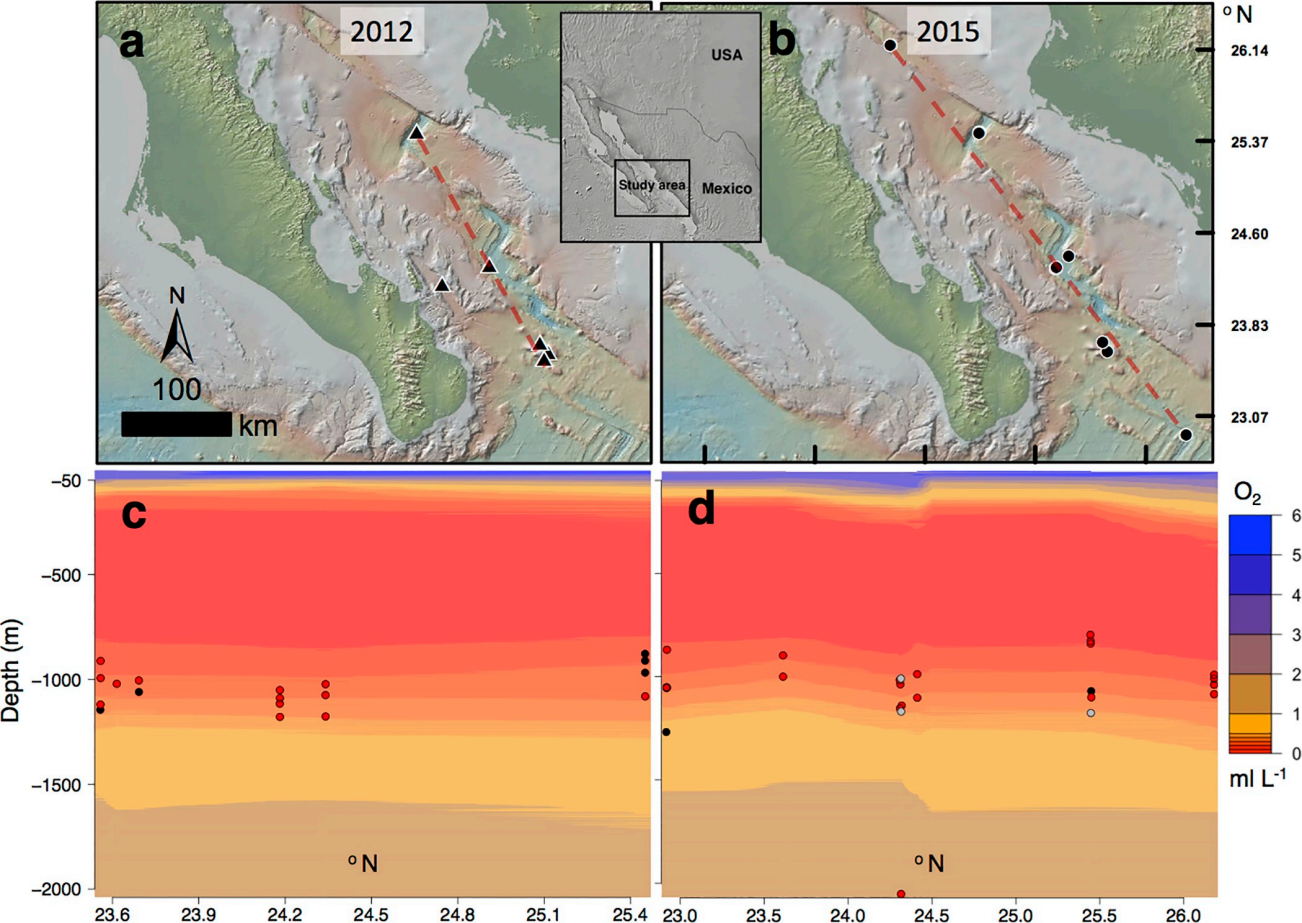
Geographic, depth, and oxygen distribution of *Hymenopenaeus doris*. Geographic, depth, and oxygen distribution of *Hymenopenaeus doris* observed by MBARI ROV *Doc Ricketts* in the Gulf of California, Mexico, during 2012 (a and c) and 2015 (b and d). Points on maps represent ROV dives where *H*. *doris* were encountered. Dashed red lines indicate approximate oxygen survey lines represented by filled contour plots of linearly interpolated ROV CTD-O data (c and d). Contour plots are overlaid with *H*. *doris* observations distinguished by time period (UTC-7): dark grey 7:00–10:00 (n = 7), light grey 10:00–14:00 (n = 3), and red 14:00–17:00 (n = 35). Most *H*. *doris* were encountered at oxygen concentrations below 0.5 ml L^-1^. S1 Fig contains analogous plots of temperature.

We recorded 45 *H*. *doris* within a depth range of 797–2072 m, between sunrise and sunset during the winters of 2012 and 2015 in the southern Gulf of California (Fig 1). Excluding one outlier, we found oxygen, temperature, and depth from each observation (±1 SD) to be 0.26 (±0.095) ml L^-1^, 4.34 (±0.35)°C, and 1036.47 (±103.29) m (S1 Table), respectively, with no difference in any parameter between years (p = 0.58) or times of day (early morning, mid-day, and evening; p = 0.13). *Hymenopenaeus doris* therefore predominantly spent the daytime hours in deep oxygen minimum zones (OMZs), with a few found in deeper, oxygen limited zones (OLZs) (Fig 1). Many pelagic crustacean species are known to undertake vertical movements during crepuscular periods or at night [17], thus it is possible that the timing of our observations prevented us from detecting shifts in vertical distribution; should they occur, vertical movements would probably be directed toward shallower, warmer waters.

**Fig 2:**
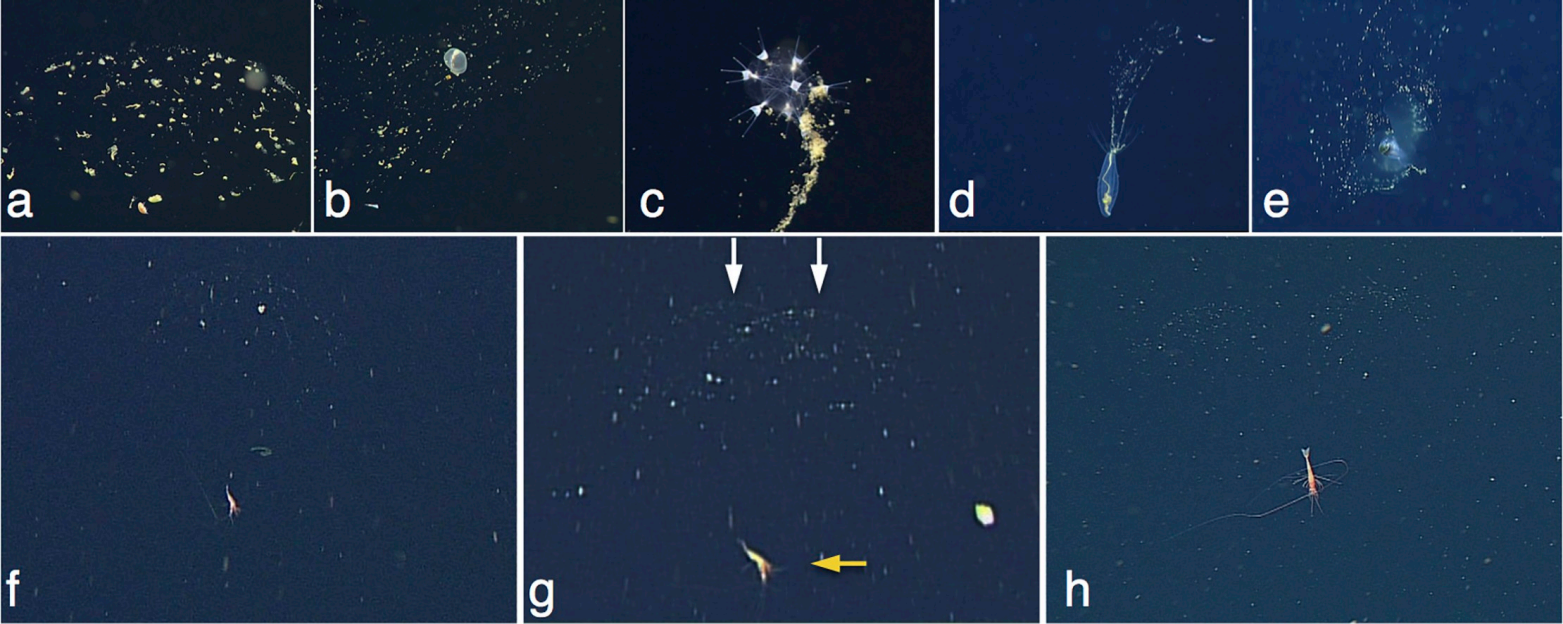
Midwater animals associated with mucous feeding webs. Examples of midwater animals that have been observed in the Monterey Canyon, California, and Gulf of California, Mexico, associated with mucous feeding webs include: amphipods (a, 2553 m), *Chaetopterus pugaporcinus* (Polychaeta) (b, 1096 m), *Tuscarantha braueri* (Phaeodaria) (c, 2388 m), *Poeobius meseres* (Polychaeta) (d, 305 m), and an undescribed pseudothecosome pteropod (e, 993 m). *Hymenopenaeus doris* has been observed three times (out of 45 total sightings) apparently situated just below a characteristically bi-lobed mucous web (f-h). However, two-dimensional imaging prevented the determination of specific animal locations with respect to webs. In (g), the orange arrow points to *H*. *doris* and the two white arrows to the associated mucous web lobes.

Numerous midwater species are detritivores [1, 31–35], and larval bivalves, larvaceans, amphipods, phaeodarians, polychaetes, and pteropods utilize mucous webs to collect sinking particles from the water column (Fig 2A–2E) [31–33, 35], a relatively passive strategy known as flux feeding [36]. We observed three out of the 45 total *H*. *doris* we encountered to be situated two to three body lengths lower than mucous webs, which consisted of two dome-shaped lobes of equal dimensions that had collected light dustings of detritus (Fig 2F–2H). For comparison, we observed *Poeobius meseres* (Fig 2D), a known flux feeder [35], during three consecutive ROV dives in the Monterey Canyon (August 2018) and found 11 out of 100 had discernable mucous webs. Based on consistencies in animal orientation with respect to the web, web size, and web structure, we hypothesize that *H*. *doris* may associate with mucous webs.

**Fig 3:**
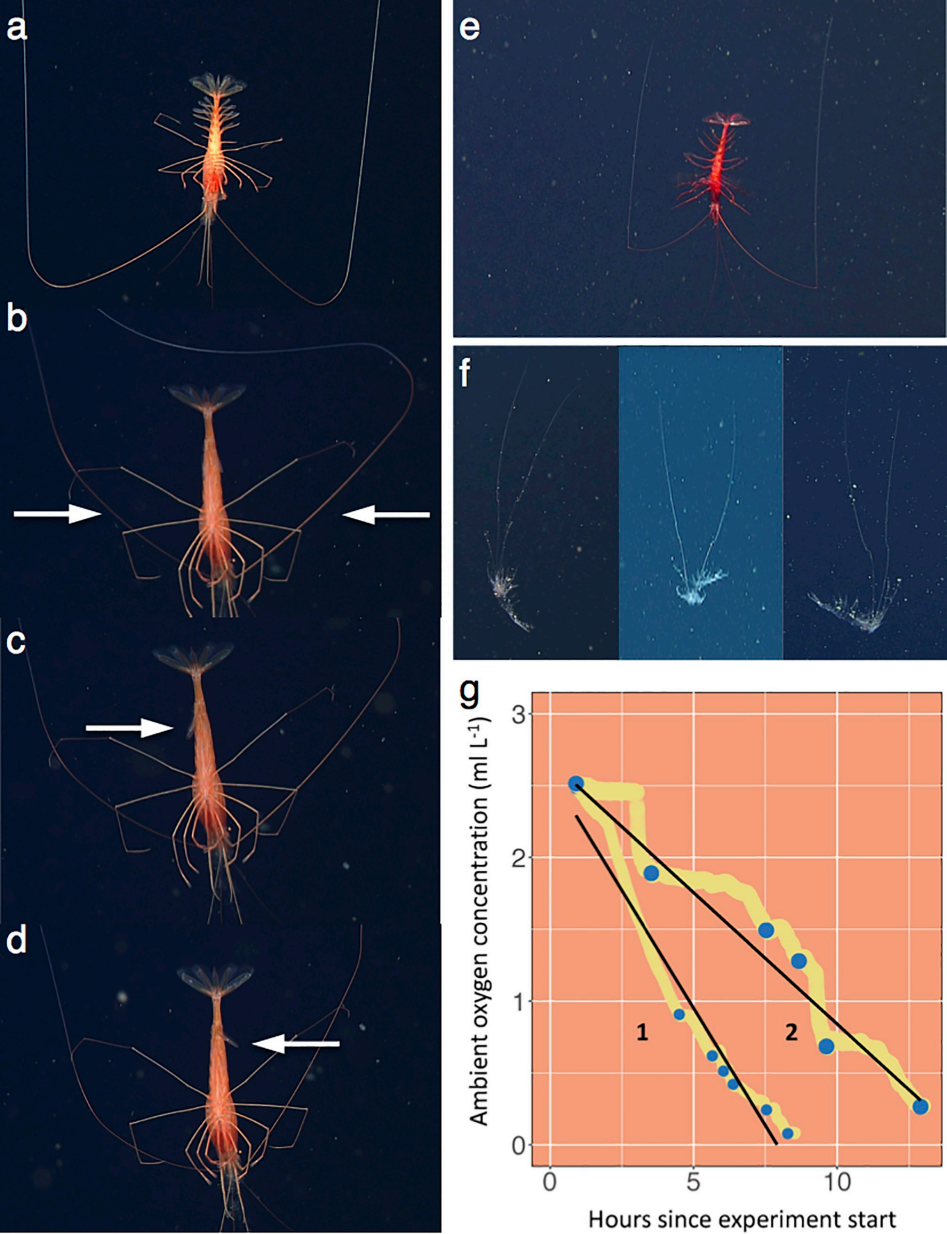
The inverted, motionless sinking behavior of two deep pelagic decapod species. The head-down, motionless sinking behavior, frequently displayed by the midwater decapod *Hymenopenaeus doris* in the Gulf of California, Mexico (a-d) and *Petalidium suspiriosum* in the Monterey Canyon, California (e), may confer visual similarity to molted shrimp exoskeletons (f). Oxygen concentration during respiration experiments of *H*. *doris* (g) declined irregularly because we relied on animal movements to mix water. In (g), blue points represent the beginning and end of periods when water was well-mixed by the animal; the slope of linear regressions (black lines) fit to these points calculated routine metabolic rates.

When observed in situ, many deep pelagic crustaceans orient head-up while steadily beating abdominal limbs (pleopods) [BPB, KLS, KRR, & BHR, pers. obs.], as is often the case in laboratory settings [17]. When initially encountered, most *H*. *doris* were instead positioned head-down, motionless, and neutral in the water column, with antennae, thoracic limbs (pereopods), and abdominal limbs (pleopods) spread laterally (Fig 3A). As we carefully approached, they reacted by slowly bending their antennae posteriorly while retracting their pleopods (Fig 3B). This allowed the animal, which is negatively buoyant, to slowly sink at a rate of ~ 1.5 m min^-1^ in a head-down orientation. Descending in this posture, *H*. *doris* appear similar to molted shrimp exoskeletons, which also sink through the midwater (Fig 3F). The shrimp maintained a consistent descent rate by controlling body orientation through minor appendage adjustments. For example, if water current from the ROV altered a shrimp’s yaw, it would react by slowly extending pleopods in the direction of the yaw shift, thereby counteracting current forces with frictional drag until the original head-down orientation was regained (Fig 3C and 3D). Through this behavior, we speculate that *H*. *doris* may visually resemble a molt—a relatively energy-poor food source. We hypothesize that this could be relevant in reducing predation.

During respiration measurements of two *H*. *doris* specimens, we relied on animal movements to circulate water in the respirometers. We calculated the routine metabolic rate of *H*. *doris* (n = 2) to range from 0.55 to 0.81 mg O_2_ kg^-1^ min^-1^ under temperatures (5°C) representing their daytime habitat (Fig 3G). This is a comparable respiration rate to other deep pelagic crustaceans predominantly inhabiting similar OMZs [3, 6]. For example, when scaled to the same experimental temperature and animal wet mass, *Neognathophausia ingens* consumed 1.33 (n = 23) and 0.67 (n = 1) mg O_2_ kg^-1^ min^-1^ at routine and basal activity levels, respectively [3].

Through similar methods, we documented another species of decapod, *Petalidium suspiriosum*, in the deep pelagic OLZ and OMZ of Monterey Canyon performing a remarkably similar head-down, motionless sinking behavior on five occasions (Fig 3E) (598–837 m, 0.24–0.32 ml L^-1^, 4.09–5.24°C). In situ observations of this second species, in addition to its capture in midwater trawls, occurred during an anomalously warm period in 2014–16 [37]. Our results suggest that *H*. *doris*, and perhaps other decapod species inhabiting analogous low-oxygen pelagic ecosystems, such as *P*. *suspiriosum*, exhibit comparable behaviors.

## Methods

### ROV specifications and observations

Our study occurred during 17–25 February 2012 and 22 February-16 March 2015, as part of the Monterey Bay Aquarium Research Institute’s (MBARI) Deep Pelagic Ecology and Biodiversity research expeditions in the Gulf of California. MBARI’s ROV *Doc Ricketts* is an electro-hydraulic vehicle outfitted with an Ikegami HDL45 HD camera, LED lights (white halogen lights in 2012), CTD-O and navigation sensors, with a depth capability from 200 to 4000 m. All ROV dives occurred between 06:00–18:00 local time, were recorded onto high-resolution 1080i D5 HD videotape, and were annotated post-dive in detail using MBARI’s Video Annotation and Reference System [38]. Observations were synchronized with ancillary metadata also logged by the ROV (CTD-O and position).

### Respiration and specimen identification

Due to its unique behavioral attributes, one particular deep pelagic crustacean was informally described as the “zombie shrimp,” in reference to the characteristic immobile, head-down posture it displayed, making it appear dead. Two of these animals were captured by ROV on 1 March 2015 in 6.5 L detritus samplers, collecting the organism and its surrounding water relatively undisturbed, for shipboard respirometry experiments and specific identification. Seawater from the collections was filtered (0.7 μm pore size, Whatman GF/F glass microfiber filter), treated with 50 mg L^-1^ each, streptomycin and ampicillin, and used for the respiration experiments. To allow for adjustment to experimental conditions, animals were kept in darkness for 19.5 hours in sample water at 5°C and then moved to cylindrical, airtight containers for experimentation. The smaller of the two specimens (0.13 g) was respired in a modified 50 ml glass syringe and the larger animal (1.19 g) in a sealed 125 ml Erlenmeyer flask (20 ml and 131 ml total experimental volume, respectively). Both chambers, as well as a seawater control, were incubated within an enclosed, insulated water bath that maintained darkness and the temperature at 5°C, conditions similar to their natural daytime environment, for the duration of the experiment. PyroScience FireStingO_2_ (http://www.pyro-science.com) and PreSence OXY-4 (http://www.presens.de) fiber-optic oxygen meters recorded oxygen concentration continuously at 15 second intervals in the 20 ml and 131 ml containers, respectively, for 16 hours. Prior to the respiration experiment, both meters were simultaneously calibrated to 0 and 100% oxygen saturation at identical temperature (10°C), salinity (34.5 psu), humidity (54.33%RH), and atmospheric pressure (1016.5 mbar) conditions. Oxygen concentrations for both animals started at ~3 ml L^-1^ and declined until they no longer consumed oxygen (0.046 and 0.27 ml L^-1^ for the small and large animal, respectively). Aside from animal movements, there was no mixing of water in the respirometry chambers.

Following the respiration analyses, the pair were frozen pending further taxonomic study by dissecting microscope. Both specimens were identified as *Hymenopenaeus doris* [22–24] and detailed morphological and behavioral characteristics were noted by examining video footage of these two animals prior to capture by the ROV. Behavioral notes were subsequently made on 43 additional encounters of *H*. *doris* annotated in the video archive from the 2012 and 2015 Gulf of California expeditions. Descriptors included body orientation, appendage position, and locomotion direction. During several occurrences, velocity (m s^-1^) was estimated by matching the descent speed of the target animal with that of the ROV. *Hymenopenaeus doris* has a distinct combination of morphological characteristics: its antennae are very long, its eyes are small and not pigmented, the carapace has small spines, its body is sparsely pigmented, and the pereopods are long and chelate [22, 23]. We note that the range of a morphologically similar species, *H*. *nereus*, has not been documented further north than 6°N [22]; the southern extent of our study area was 22.9°N, thus, we have confidence in the in situ identifications of *H*. *doris* that were observed but not collected.

### Monterey Canyon ROV and trawl surveys

In July, August, and December 2015, as well as December 2016, Tucker trawls operated off MBARI’s R/V *Western Flyer* sampled the nighttime Monterey Canyon midwater zooplankton community. In addition to other decapods, *Petalidium suspiriosum* was identified in trawls operating from 0–1100 m. Its body is mostly deep red, with a dark purplish region in the anterior thorax and a black pigment fleck on the dorsal surface of the ocular segment between the eyes. Similar to *H*. *doris*, *P*. *suspiriosum* has elongate antennae and pereopods. However, its pereopods have more setae and its eyes are pigmented [39]. Recent revision of the genus *Petalidium* maintains that *P*. *suspiriosum* is the only North Pacific species [40]. During June 2016, one *P*. *suspiriosum* was collected and identified from an ROV dive in Monterey Canyon. In a similar fashion to the in situ observations of *H*. *doris*, the ROV footage of *P*. *suspiriosum* was examined for notable morphological and behavioral characteristics. Behavioral notes were subsequently made on four additional encounters with *P*. *suspiriosum* in Monterey Canyon found by searching MBARI’s video archive.

### Statistical analysis

All data analyses and figures were made in R [41]; ArcMap and Photoshop were also used for figure construction. Processed ROV CTD-O from the Gulf of California, excluding two ROV dives in 2012 and 2015 due to limited dive duration and depth, were linearly interpolated and used in conjunction with geographic data to construct filled contour plots of oxygen concentration (Fig 1). A Type II MANOVA with a Pillai–Bartlett post-hoc test was used to compare distribution parameters recorded during each observation of *H*. *doris* (depth, oxygen, temperature) (S1 Table) between year (2012, 2015) and time of day (07:00–10:00, 10:00–14:00, 14:00–17:00).

Routine metabolic rates were calculated for a 0.13 g (1; Fig 3G) and a 1.19 g (2; Fig 3G) *H*. *doris* from the slope of linear regressions fit to oxygen concentration values at the beginning and end of activity periods, excluding the first hour of experiments (Fig 3G); rates were scaled to an animal of intermediate size (0.66 g) using a quarter-power metabolic scaling relationship [42] in the absence of any species-specific data. Reference [3] reports the mass-specific routine metabolic rate of *Neognathophausia ingens* ranging from 0.3–15 g wet weight at 5.5°C during regular swimming to be 0.7 mg O_2_ kg^-1^ wet mass min^-1^ (n = 23), and basal metabolic rates during inactivity to be 0.4 mg O_2_ kg^-1^ wet mass min^-1^ (n = 1). Assuming that the average wet weight of these specimens was 7.35 g, the routine and basal metabolic rates of *N*. *ingens* are 1.33 and 0.67 mg O_2_ kg^-1^ wet mass min^-1^, when scaled to 0.66 g using quarter-power scaling and adjusted to 5°C using a temperature scaling relationship with a Q10 of 2.5.

### Research ethics

All methods were carried out in accordance with relevant guidelines and regulations.

## Supporting information

S1 Table Metadata associated with shrimp observations. (PDF)

S1 Fig Geographic, depth, and temperature distribution of *Hymenopenaeus doris*. Geographic, depth, and temperature distribution of *Hymenopenaeus doris* observed by MBARI ROV *Doc Ricketts* in the Gulf of California, Mexico, during 2012 (a and c) and 2015 (b and d). Points on maps represent ROV dives where *H*. *doris* were encountered. Dashed red lines indicate approximate temperature survey lines represented by filled contour plots of linearly interpolated ROV CTD-O data (c and d). Contour plots are overlaid with *H*. *doris* observations distinguished by time period (UTC-7): dark grey 7:00–10:00 (n = 7), light grey 10:00–14:00 (n = 3), and red 14:00–17:00 (n = 35). Most *H*. *doris* were encountered from 4–5°C.
(TIFF)
