# Supplementary material for: Pelagic shrimp play dead in deep oxygen minima
Source: PLoS One. 2018 Nov 28;13(11):e0207249. doi: 10.1371/journal.pone.0207249 (PMC6261571; doi:10.1371/journal.pone.0207249)
Supplement: S1 Table — (PDF) [file pone.0207249.s001.pdf]

Table S1. Metadata associated with shrimp observations.

H. doris	*Dive*	*Date*	*GMT*	*Latitude*	*Longitude*	*Depth*	*Oxygen*	*Temp*	*Salinity*
1	D0333-02	2/17/12	21:30:55	24.181681	-109.633076	1051	0.266	4.336	34.535
2	D0333-02	2/17/12	21:42:03	24.181306	-109.633105	1116	0.329	4.137	34.544
3	D0333-04	2/17/12	23:52:34	24.181863	-109.633032	1090	0.286	4.286	34.538
4	D0333-04	2/17/12	23:45:37	24.181877	-109.633136	1179	0.38	4.004	34.55
5	D0336-07	2/19/12	21:25:22	23.61506	-108.749128	1021	0.233	4.446	34.533
6	D0337-01	2/20/12	14:50:00	23.557998	-108.783125	1145	0.383	3.994	34.55
7	D0337-10	2/20/12	23:27:45	23.559068	-108.782314	994	0.219	4.486	34.534
8	D0337-10	2/20/12	23:14:24	23.558589	-108.782444	1120	0.348	4.082	34.546
9	D0337-10	2/20/12	23:42:27	23.558954	-108.782391	912	0.162	4.746	34.524
10	D0339-02	2/21/12	17:14:46	23.692068	-108.817492	1060	0.246	4.373	34.534
11	D0339-06	2/21/12	22:06:18	23.691761	-108.817806	1005	0.218	4.481	34.532
12	D0341-01	2/23/12	14:34:36	25.450449	-109.849602	878	0.175	4.701	34.531
13	D0341-01	2/23/12	14:35:42	25.450542	-109.849553	911	0.203	4.526	34.539
14	D0341-01	2/23/12	14:37:39	25.450666	-109.849481	968	0.227	4.398	34.544
15	D0341-10	2/23/12	23:31:08	25.450973	-109.848023	1081	0.287	4.092	34.556
16	D0343-09	2/25/12	22:47:28	24.340471	-109.241735	1177	0.394	3.977	34.554
17	D0343-10	2/25/12	23:07:01	24.340239	-109.241964	1075	0.29	4.255	34.545
18	D0343-10	2/25/12	23:17:09	24.340411	-109.241696	1023	0.238	4.433	34.537
19	D0713-04	2/25/15	19:32:29	25.446591	-109.849541	1176	0.345	3.903	34.564
20	D0715-10	2/27/15	22:06:45	26.180837	-110.591563	1010	0.179	4.468	34.546
21	D0715-10	2/27/15	22:08:23	26.180955	-110.59151	991	0.159	4.57	34.546
22	D0715-10	2/27/15	22:02:55	26.180609	-110.591456	1039	0.187	4.371	34.55
23	D0715-10	2/27/15	21:56:58	26.180676	-110.590656	1085	0.248	4.212	34.553
24	D0717-03	3/1/15	19:45:36	24.316412	-109.199707	1169	0.413	3.894	34.556
25	D0717-04	3/1/15	20:20:39	24.315342	-109.201464	1009	0.215	4.446	34.534
26	D0717-06	3/1/15	21:48:14	24.311561	-109.204786	1037	0.254	4.315	34.539
27	D0717-07	3/1/15	22:50:56	24.308969	-109.209011	1154	0.373	3.97	34.552
28	D0717-08	3/1/15	23:40:12	24.307573	-109.211557	1017	0.227	4.408	34.537
29	D0718-12	3/2/15	23:14:55	24.412111	-109.09687	1102	0.332	4.08	34.548
30	D0718-13	3/3/15	0:08:17	24.412037	-109.097058	988	0.211	4.456	34.534
31	D0722-12	3/8/15	23:36:39	25.44926	-109.843359	1100	0.321	4.075	34.552
32	D0723-02	3/9/15	14:51:51	25.448357	-109.848327	1070	0.281	4.189	34.547
33	D0723-11	3/9/15	22:48:26	25.444817	-109.849862	840	0.087	5.111	34.525
34	D0723-11	3/9/15	22:49:41	25.444718	-109.849878	828	0.084	5.132	34.525
35	D0723-11	3/9/15	23:09:30	25.443694	-109.85077	797	0.065	5.395	34.521
36	D0724-09	3/10/15	21:17:13	24.315865	-109.199634	2072	1.706	2.141	34.641
37	D0724-12	3/10/15	23:43:09	24.319209	-109.199591	1140	0.334	4.047	34.551
38	D0725-10	3/11/15	21:46:10	23.612609	-108.768895	1000	0.298	4.155	34.542
39	D0725-10	3/11/15	21:56:09	23.612487	-108.770083	897	0.176	4.547	34.53
40	D0727-02	3/13/15	14:54:20	22.916796	-108.115725	1268	0.565	3.563	34.567
41	D0727-12	3/13/15	23:28:35	22.9193	-108.113025	1052	0.269	4.235	34.539
42	D0727-12	3/13/15	23:31:10	22.91922	-108.112905	1054	0.266	4.231	34.537
43	D0727-12	3/13/15	23:31:17	22.919213	-108.112911	1054	0.27	4.222	34.541
44	D0727-12	3/13/15	23:30:32	22.919269	-108.112921	1052	0.269	4.231	34.539
45	D0727-12	3/13/15	0:02:54	22.920724	-108.113503	870	0.145	4.859	34.51
P. suspiriosum									
1	D0598-01	4/28/14	14:25:00	36.535369	-122.508753	598	0.235	5.241	34.361
2	D0602-06	5/1/14	18:59:15	36.748989	-122.09544	837	0.324	4.523	34.401
3	V3863-03	9/11/15	18:37:56	36.698381	-122.05584	803	0.298	4.649	34.377
4	V3981-06	12/8/15	21:35:57	36.701072	-122.061397	740	0.214	4.982	34.314
5	D0856-06	6/11/16	18:56:03	36.33965	-122.30454	829	0.305	4.093	34.432
