# Supplementary figures and images for: Pelagic shrimp play dead in deep oxygen minima
Source: PLoS One. 2018 Nov 28;13(11):e0207249. doi: 10.1371/journal.pone.0207249 (PMC6261571; doi:10.1371/journal.pone.0207249)

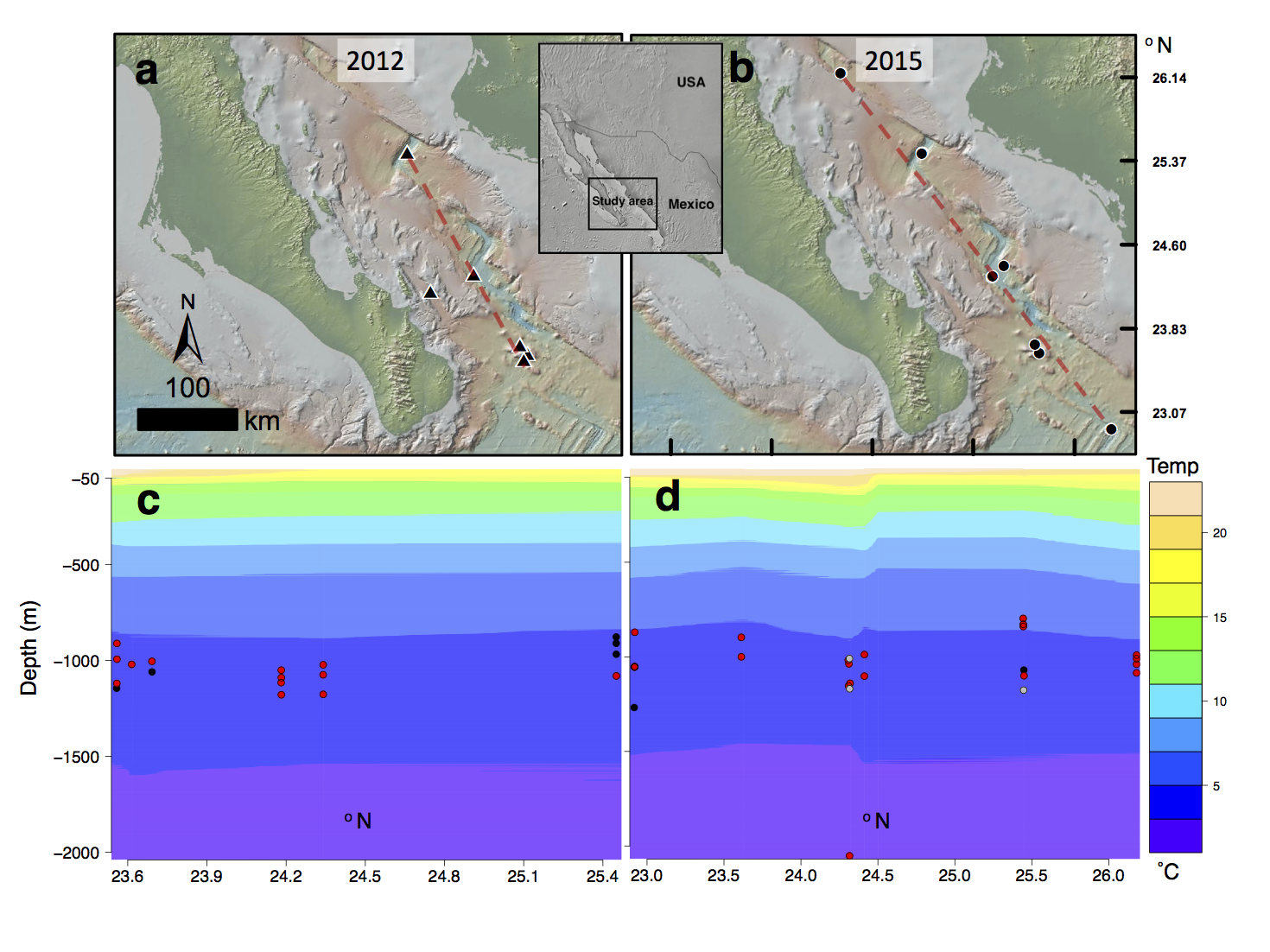

Supplement: S1 Fig — Geographic, depth, and temperature distribution of Hymenopenaeus doris observed by MBARI ROV Doc Ricketts in the Gulf of California, Mexico, during 2012 (a and c) and 2015 (b and d). Points on maps represent ROV dives where H. doris were encountered. Dashed red lines indicate approximate temperature survey lines represented by filled contour plots of linearly interpolated ROV CTD-O data (c and d). Contour plots are overlaid with H. doris observations distinguished by time period (UTC-7): dark grey 7:00–10:00 (n = 7), light grey 10:00–14:00 (n = 3), and red 14:00–17:00 (n = 35). Most H. doris were encountered from 4–5°C. (TIFF) [file pone.0207249.s002.tiff]
